# Substrate Microarchitecture Shapes the Paracrine Crosstalk of Stem Cells with Endothelial Cells and Osteoblasts

**DOI:** 10.1038/s41598-017-15036-x

**Published:** 2017-11-09

**Authors:** Francisco Martín-Saavedra, Lara Crespo, Clara Escudero-Duch, Laura Saldaña, Enrique Gómez-Barrena, Nuria Vilaboa

**Affiliations:** 1 CIBER de Bioingeniería, Biomateriales y Nanomedicina (CIBER-BBN), Madrid, Spain; 2 Hospital Universitario La Paz-IdiPAZ, Paseo de la Castellana 261, 28046 Madrid, Spain; 3 Departamento de Cirugía, Universidad Autónoma de Madrid, Calle del Arzobispo Morcillo 4, 28029 Madrid, Spain

## Abstract

We examined the hypothesis that substrate microarchitecture regulates the crosstalk between human mesenchymal stem cells (hMSC) and cell types involved in bone regeneration. Compared with polyester flat substrates having uniformly distributed homogenous pores (2D), three-dimensional polystyrene substrates with randomly oriented and interconnected pores of heterogeneous size (3D) stimulated the stromal secretion of IGF-1 while lessened the production of VEGFR-1, MCP-1 and IL-6. The medium conditioned by hMSC cultured in 3D substrates stimulated tube formation by human endothelial cells (hEC) to a higher extent than medium from 2D cultures. 3D co-cultures of hMSC and hEC contained higher secreted levels of IGF-1, EGF and FGF-2 than 2D co-cultures, resulting in increased hEC proliferation and migration. Substrate microarchitecture influenced the secretion of factors related to bone remodeling as the ratio RANKL to OPG, and the levels of M-CSF and IL-6 were higher in 3D co-cultures of hMSC and human osteoblasts (hOB) than in 2D co-cultures. Cytokine microenvironment in 3D co-cultures stimulated osteoblast matrix reorganization while demoted the late steps of osteoblastic maturation. Altogether, data in this study may unveil a new role of scaffold microarchitecture during bone regeneration, as modulator of the paracrine relationships that hMSC establish with hEC and hOB.

## Introduction

Due to their self-renewal and multilineage differentiation potential, mesenchymal stem cells (MSC) emerged as extremely attractive tools for cell-based transplantation therapies and tissue engineering applications. MSC can be easily cultured and expanded *in vitro*, which has facilitated preclinical studies and the launch of clinical trials which are rendering promising results. While MSC can differentiate *in vivo* into chondrocytes, osteoblasts, adipocytes, myocytes or cardiomyocytes, among other cell types, the precise mechanisms that underlie their therapeutic activities are not well understood. In fact, survival and engraftment rates of transplanted MSC are typically very low, thereby remaining active during a short time window, and delivery of MSC or media conditioned by these cells often result in similar beneficial outcomes^1–3^. These observations led to the shift of a paradigm centered on their progenitor function to other based on the paracrine control that MSC exert on other cell types^4^. Available data support the view that transplanted MSC establish coordinated interactions with their local environment, acting as factories of trophic, anti-fibrotic, immunomodulatory and chemoattractant factors that play a predominant role during tissue regeneration and repair^5^.

Bone tissue engineering aims to develop substrates that establish proper interactions with MSC to generate *ex vivo* “tissue intermediates” which, upon implantation in the injured bone site, unlock mechanisms of self-regeneration. While transplanted MSC may act as progenitors of newly formed bone^6^, several studies reported that the regenerated tissue derives mainly^7^ or even entirely^8,9^ from host progenitors, supporting the view that donor MSC behave as inducers rather than effectors of bone regeneration. Short-lived transplanted MSC effectively establish paracrine interactions with host cells, mediating the mobilization of macrophages, as well as of osteogenic and endothelial progenitors into the implant as shown in experimental models of ectopic and orthotopic bone formation^10–12^. In conjunction with cell-to-cell contact, crosstalk between donor MSC and host cells contributes to establish proper structural and functional connections between the engineered tissue intermediate and the host bone. Thus, the building of vascularized networks within the implanted tissue intermediate is supported by the paracrine interactions that MSC establish with host endothelial cells (EC)^13^. *In vitro* studies have revealed relationships between MSC and OB in the absence of direct cell-to-cell contact^14,15^, reinforcing the hypothesis that bone regeneration is elicited by paracrine signalling between transplanted MSC and host bone-forming cells.

The physicochemical characteristics of the substrates have a decisive influence on the dynamics of MSC secretion^16^. In turn, the paracrine control that host cells exert on donor MSC may be also tuned by substrate features. We have recently reported that the microarchitecture of the substrates that harbour MSC influence their cross-talk with macrophages^17^. Interestingly, levels of soluble factors related with inflammation and chemotaxis are substantially lower in co-cultures of human macrophages and hMSC seeded in polystyrene substrates with randomly oriented and interconnected pores of variable size than in co-cultures of hMSC seeded on polyester flat surfaces with uniform porosity. Both types of substrates, named 3D and 2D respectively, were used in the present study to investigate to what extent their topographical features might influence the paracrine relationships that transplanted hMSC maintain with resident cells that participate in bone healing, such as hEC or hOB.

## Results

### Cell organization on 2D and 3D substrates

**Figure 1:**
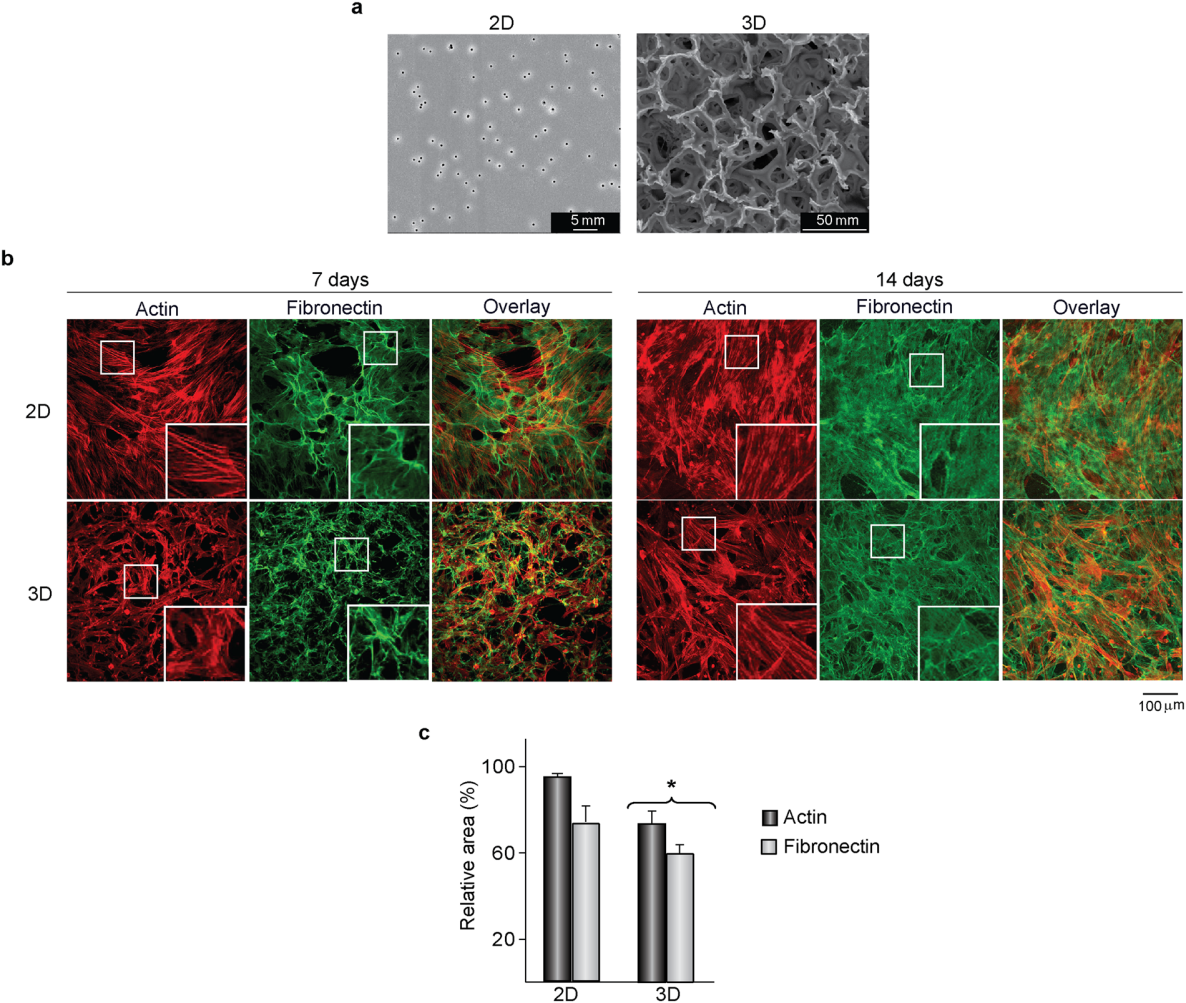
Actin cytoskeleton and fibronectin matrix organization in hMSC cultured on 2D or 3D substrates. (**a**) SEM images of 2D and 3D substrates. (**b**) Confocal images showing double staining of actin and fibronectin in cells cultured on the substrates for 7 and 14 days. Boxed areas showing 2.5x magnified images (**c**) Relative image areas occupied by actin and fibronectin in cultures incubated for 7 days. *p < 0.05 compared with 2D cultures.

hMSC were cultured on the substrates shown in Fig. 1a for 7 days, and then doubled stained for actin and fibronectin (Fig. 1b). On polyester 2D substrates, cells adopted a flat, spindle-shaped morphology, with well-developed actin bundles extended through the cell body. Actin filaments arranged in more closely package arrays in cells growing in 3D substrates, as revealed by quantification of the image areas occupied by stained cytoskeleton (Fig. 1c). Also, the interconnected network of fibronectin fibrils was more compacted in 3D than in 2D substrates (Fig. 1c). After culturing for 14 days, hMSC reached confluency on both substrates and displayed a well-spread morphology with a similarly well-organized actin cytoskeleton and a dense fibronectin matrix covering the cell layer.

### The crosstalk between hMSC and HUVEC is regulated by substrates features

**Figure 2:**
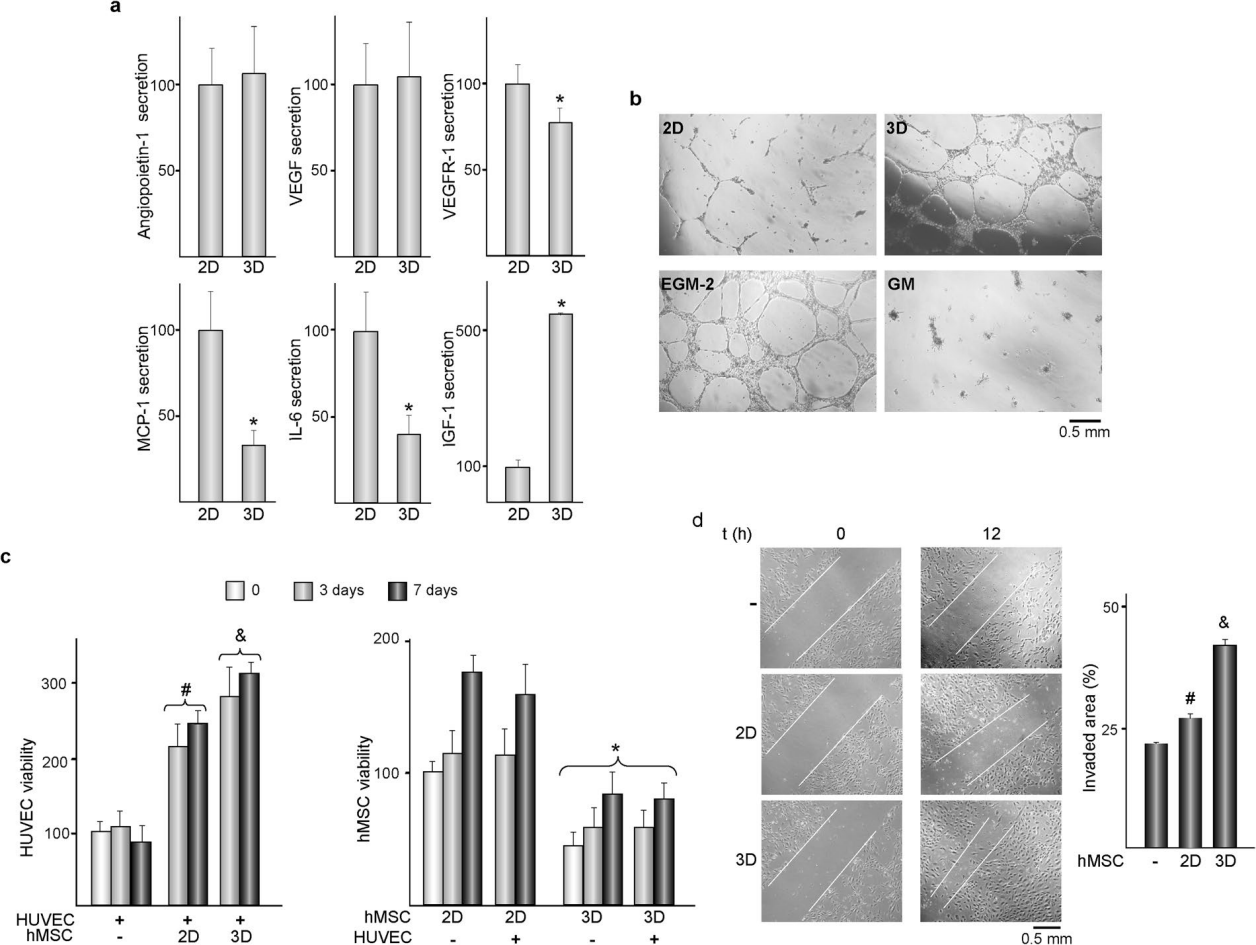
2D and 3D substrates differentially affect the paracrine relationships that hMSC establish with HUVEC. (**a**) Secreted angiopoietin-1, VEGF, VEGFR-1, MCP-1, IL-6 and IGF-1 levels in media conditioned by hMSC cultured for 7 days on 2D or 3D substrates. Data are relative to the levels measured in 2D cultures, which were given an arbitrary value of 100 and correspond to the values shown in the Table 1. (**b**) Representative phase contrast micrographs of HUVEC incubated in media conditioned by hMSC cultured on 2D or 3D substrates, in EGM-2 or in growth medium (GM). (**c**) Metabolic activity of HUVEC or hMSC. HUVEC were co-cultured up to 7 days with hMSC seeded on 2D or 3D substrates. (−) and (+) indicate the absence or presence of the corresponding cell type, respectively. Data are relative to the metabolic activities of isolated-cultures of HUVEC or hMSC seeded on 2D substrates, which were measured immediately before setting the co-cultures (0), and were given an arbitrary value of 100. (**d**) Left panel: representative phase contrast micrographs of HUVEC monolayers scratched and then incubated in GM (−) or co-cultured with hMSC seeded on 2D or 3D substrates. Images were taken immediately after scratching (0) and 12 h later. White lines mark the edge of the “wound”. Right panel: Percentage of invaded areas. *p < 0.05 compared with hMSC seeded on 2D substrates under the same experimental condition. ^#^p < 0.05 compared with isolated cultures of HUVEC. ^&^p < 0.05 compared with co-cultures of HUVEC and hMSC seeded on 2D substrates, under the same experimental condition.

Next, we investigated whether several soluble factors which may influence human umbilical vein EC (HUVEC) behaviour are differentially secreted by hMSC cultured for 7 days on flat or three-dimensional substrates (Fig. 2a). No differences were detected in the secretion of angiopoietin-1 or vascular endothelial growth factor (VEGF). However, levels of soluble receptor-1 of VEGF (VEGFR-1), monocyte chemotactic protein-1 (MCP-1) and interleukin-6 (IL-6) were lower in 3D cultures while insulin-like growth factor (IGF-1) increased by about 5-fold. Fibroblast growth factor 2 (FGF-2) and epidermal growth factor (EGF) could not be detected in hMSC cultured on any substrate. We then asked whether tube-like structures are differentially generated in hEC cultures incubated in growth media conditioned by hMSC seeded on flat or three-dimensional substrates (Fig. 2b). Tube formation assays showed that HUVEC cultured with medium conditioned by hMSC cultured in 3D substrates self-assembled and elongated, forming a capillary-like network with typical closed structures similar to the observed in HUVEC cultured in endothelial cell growth medium. Incubation of HUVEC with medium conditioned by hMSC cultured on 2D substrates resulted in shortened, much narrower and open tube networks, while fresh growth medium failed to rearrange endothelial cells in tubular-like structures.

**Figure 3:**
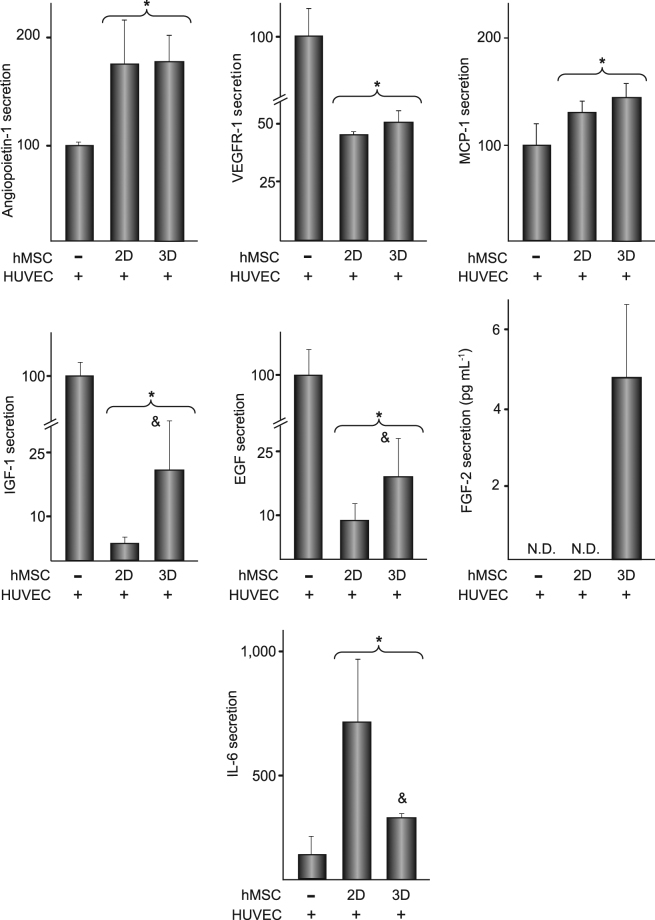
Angiopoietin-1, VEGFR-1, MCP-1, IGF-1, EGF, FGF-2 and IL-6 secretion in co-cultures of HUVEC and hMSC. HUVEC were cultured in isolation (−) or co-cultured with hMSC seeded on 2D or 3D substrates for 7 days. Except for FGF-2, data are relative to the levels of isolated cultures of HUVEC, which were given an arbitrary value of 100 and correspond to the values shown in the Table 1. *p < 0.05 compared with isolated cultures of HUVEC. ^&^p < 0.05 compared with co-cultures of HUVEC and hMSC seeded on 2D substrates. N.D.: Not detected.

A second set of experiments were conducted to investigate whether cell viability and migration of HUVEC were modulated when co-cultured up to 7 days with hMSC seeded on 2D or 3D substrates. Metabolic activity of isolated cultures of HUVEC remained unchanged with time when incubated in growth medium (Fig. 2c, left panel), but was highly stimulated when co-cultured with hMSC. The increase was more pronounced in 3D than in 2D co-cultures. Metabolic activity of hMSC cultured in isolation was higher in 2D than in 3D substrates and increased with culture time in both conditions (Fig. 2c, right panel). Regardless of the type of substrate, co-culturing with HUVEC did not affect the metabolic activity of hMSC. HUVEC migratory activity increased when co-cultured with hMSC (Fig. 2d, left panel). Quantification of invaded area indicated that wound closure was notably higher in co-cultures with hMSC seeded in 3D than on 2D substrates (Fig. 2d, right panel). We then assessed whether the levels of the soluble factors previously examined in isolated cultures of hMSC grown on 2D or 3D substrates (Fig. 2a) are differentially modulated in co-cultures. Angiopoietin-1 was secreted at lower rate by HUVEC than by hMSC (Table 1). Its levels were higher in co-cultures than in HUVEC cultured in isolation (Fig. 3), and both types of co-cultures showed a similar secretion rate. VEGF could not be detected neither in isolated cultures of HUVEC nor in co-cultures. HUVEC secreted higher levels of VEGFR-1, MCP-1, IGF-1 and EGF than hMSC (Table 1). Co-cultures media contained substantially lower levels of VEGFR-1, IGF-1 and EGF than media from isolated cultures of HUVEC, while MCP-1 levels increased (Fig. 3). Levels of VEGFR-1 and MCP-1 were similar in 2D and 3D co-cultures. However, concentrations of IGF-1 and EGF were higher in co-cultures with hMSC grown in substrates with interconnected pores. FGF-2 was detected in 3D but not in 2D co-cultures, nor in isolated cultures (Fig. 3). Finally, we examined the levels of IL-6, secreted at lower extent by HUVEC than by hMSC (Table 1). Compared with isolated cultures of HUVEC, co-cultures contained higher cytokine concentration, an effect that was enhanced in 2D co-cultures (Fig. 3).

**Table 1:** Levels of soluble factors in media conditioned by HUVEC or hMSC cultured for 7 days on 2D substrates.

	HUVEC	hMSC
Angiopoietin-1	0.97 ± 0.16	1.71 ± 0.36^*^
VEGF	ND	3.92 ± 0.9
VEGFR-1	5.87 ± 0.92	0.23 ± 0.03^*^
MCP-1	12.28 ± 2.79	4.26 ± 1.00^*^
IL-6	3.15 ± 2.24	11.83 ± 2.80^*^
IGF-1	1.31 ± 0.1	0.14 ± 0.06^*^
FGF-2	N.D.	N.D.
EGF	0.07 ± 0.01	N.D.

The data are expressed as ng ml^−1^ of culture medium. Each value represents the mean ± S.D. of five independent experiments. *p < 0.05 compared with cultures of HUVEC. N.D.: not detected.

### The crosstalk between hMSC and hOB is regulated by substrate features

**Figure 4:**
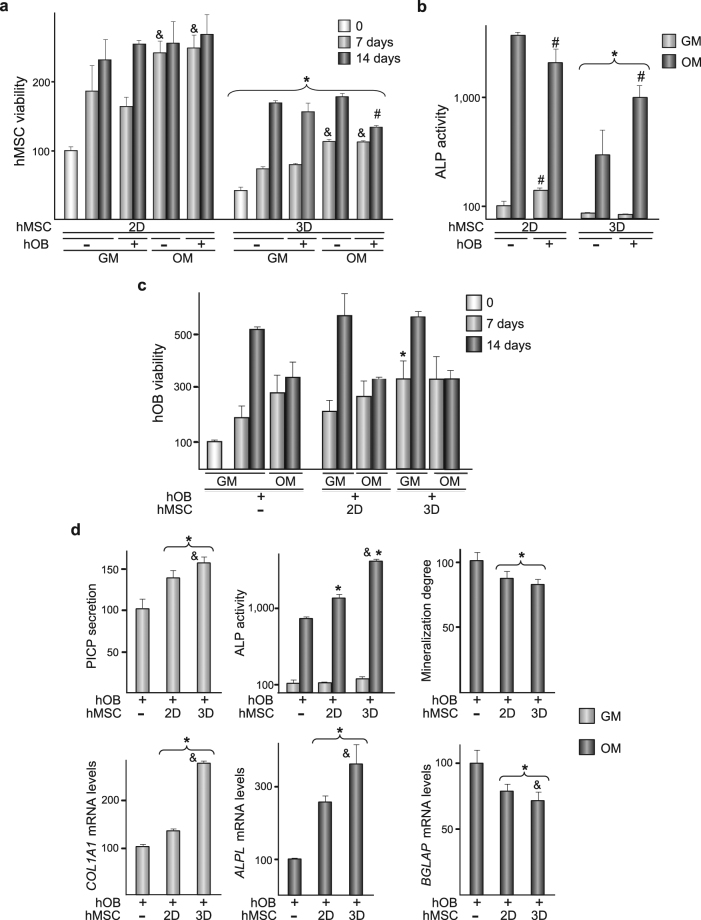
2D and 3D substrates differentially affect the paracrine relationships that hMSC establish with hOB. (**a**,**b**) hMSC seeded on 2D or 3D substrates were cultured in isolation (−) or co-cultured with hOB (+) in growth (GM) or osteogenic (OM) media up to 14 days. (**a**) hMSC viability. Data are relative to the viability of isolated cultures of hMSC seeded on 2D surfaces which was measured immediately before setting the co-cultures (0), and was given an arbitrary value of 100. (**b**) ALP activity in hMSC. Data are relative to the levels of isolated cultures of hMSC grown in GM on 2D substrates, which were given an arbitrary value of 100 and correspond to 5 ± 2 nmol *p*-nitrophenol min^−1^ mg protein^−1^. *p < 0.05 compared with hMSC seeded on 2D substrates, under the same experimental condition. ^#^p < 0.05 compared with isolated cultures of hMSC, under the same experimental condition. ^&^p < 0.05 compared with hMSC cultured in GM, under the same experimental condition. (**c,d**) hOB were cultured in isolation (−) or co-cultured with hMSC seeded on 2D or 3D substrates up to 14 days, in GM or OM. (**c**) hOB viability. Data are relative to the viability of isolated cultures of hOB which was measured immediately before setting the co-cultures (0), and was given an arbitrary value of 100. (**d**) Upper panel: PICP levels in co-cultures incubated in GM, ALP activity in hOB co-cultured in GM or OM, and relative degree of cell layer mineralization in hOB co-cultured in OM. PICP data are relative to the levels of isolated cultures of hOB, which were given an arbitrary value of 100 and correspond to the value shown in Table 2. ALP activity data are relative to the levels of isolated cultures of hOB incubated in GM, which were given an arbitrary value of 100 and correspond to 16 ± 6 nmol *p*-nitrophenol min^−1^ mg protein^−1^. Lower panel: Relative *COL1A1*, *ALPL* and *BGLAP* mRNA levels in hOB co-cultured as in the upper panel. *p < 0.05 compared with isolated cultures of hOB. ^&^p < 0.05 compared with hOB co-cultured with hMSC seeded on 2D substrates.

First, we measured the metabolic activities of hMSC seeded on 2D or 3D substrates and cultured in isolation or co-cultured with hOB, in growth or osteogenic media (Fig. 4a). Detected activities increased in both media during the observation period, and were higher in hMSC cultured on 2D than in 3D substrates. Metabolic activity of hMSC cultured in osteogenic medium for 7 days was higher than in cells cultured in growth medium, although no differences were detected when the incubation was prolonged to 14 days. Similar effects were observed in hMSC co-cultured with hOB. No differences were detected between hMSC cultured in isolation or co-cultured with hOB except in co-cultured hMSC that had been grown in 3D substrates for 14 days in osteogenic medium, which experienced a decrease of metabolic activity. Next, we measured alkaline phosphatase (ALP) activities in hMSC cultured on the substrates in isolation or co-cultured with hOB for 14 days, in growth or osteogenic medium (Fig. 4b). ALP activities were induced in osteogenic medium, and were higher in hMSC cultured on 2D than in 3D substrates. However, co-culturing with hOB decreased the extent of the induction in hMSC grown on 2D substrates while substantially increased it in 3D conditions.

Metabolic activity of isolated cultures of hOB incubated in growth medium increased during the 2-week observation period, and was similar in hOB co-cultured with hMSC seeded on  2D substrates (Fig. 4c). Co-culturing for 7 days with hMSC seeded on 3D substrates increased hOB viability, while no differences were observed when the incubation period was prolonged. When cultured on 2D substrates for 2 weeks, hOB released higher amounts of pro-collagen I α1 (PICP) than hMSC (Table 2). As compared with isolated cultures of hOB, levels of the propeptide increased in co-cultures (Fig. 4d, upper-left panel). Despite no differences were detected between the secreted levels of PICP in hMSC cultured on 2D or 3D substrates (data not shown), propeptide levels were higher in 3D co-cultures. PICP secretion data correlated well with changes in mRNA levels of osteoblastic *COL1A1* gene (Fig. 4d, lower-left panel). When cultures were incubated in osteogenic medium, hOB viability increased during the first week, remaining unchanged afterwards, and was not affected by co-culturing with hMSC incubated on 2D or 3D substrates (Fig. 4c). However, induced ALP activity in hOB incubated in osteogenic medium for 2 weeks increased when they were co-cultured with hMSC (Fig. 4d, upper-middle panel). Stimulation of the enzymatic activity was more prominent in 3D co-cultures. *ALPL* mRNA accumulation in hOB incubated in osteogenic medium followed the same trend than enzymatic activity (Fig. 4d, lower-middle panel). Matrix mineralization decreased when hOB were co-cultured with hMSC in osteogenic medium (Fig. 4d, upper-right panel), an effect not influenced by the type of substrate. Osteoblastic *BGLAP* mRNA levels decreased by co-culturing with hMSC, being the decrease more pronounced in 3D co-cultures (Fig. 4d, lower-right panel).

**Table 2:** Levels of soluble factors in  media conditioned by hOB or hMSC cultured for 14 days on 2D substrates.

	hOB	hMSC
PICP	(189.00 ± 23.00) × 10^3^	(59.19 ± 5.18) × 10^3^*
RANKL	5.01 ± 1.43	98.27 ± 15.66*
OPG	(5.94 ± 1.39) × 10^3^	94.52 ± 42.63*
M-CSF	134.13 ± 84.16	48.86 ± 9.77*
IL-6	(236.19 ± 62.69) × 10^3^	(83.06 ± 17.60) × 10^3^*
VEGF	(13.56 ± 0.81) × 10^3^	(10.00 ± 3.72) × 10^3^
VEGFR-1	17.85 ± 3.55	5.98 ± 2.67*

The data are expressed as pg ml^−1^ of culture medium. Each value represents the mean ± S.D. of five independent experiments. *p < 0.05 compared with cultures of hOB.

**Figure 5:**
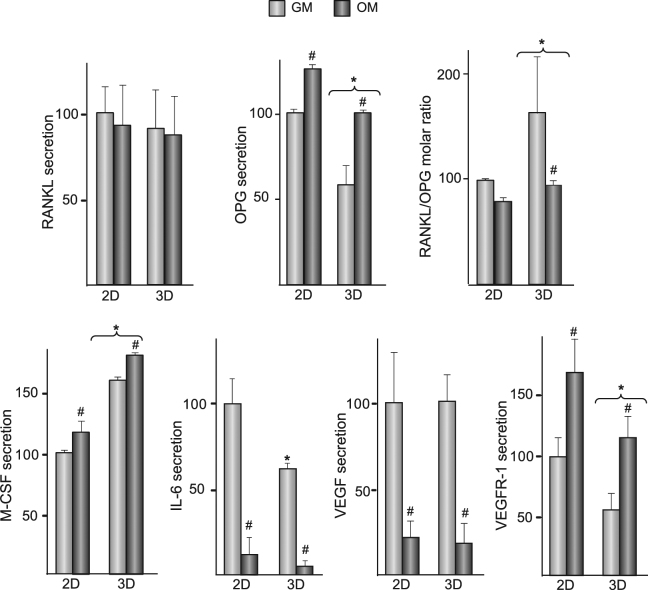
RANKL, OPG, M-CSF, IL-6, VEGF and VEGFR-1 secretion in hMSC cultured on 2D or 3D substrates. hMSC were seeded on 2D or 3D substrates and cultured for 14 days in growth (GM) or osteogenic (OM) media. Protein levels are relative to those measured in hMSC seeded on 2D substrates and cultured in GM, which were given an arbitrary value of 100 and correspond to the values shown in the Table 2. *p < 0.05 compared with hMSC seeded on 2D substrates, under the same experimental condition. ^#^p < 0.05 compared with hMSC cultured in GM.

To investigate whether soluble factors that influence bone remodelling are differentially secreted in hMSC cultured on flat or three-dimensional substrates, cells were cultured on the materials for 14 days, in growth or osteogenic media (Fig. 5). Regardless the composition of the media, the levels of the soluble form of the receptor activator of nuclear factor kappa-B ligand (RANKL) remained unchanged in 2D and 3D cultures. In both substrates, osteoprotegerin (OPG) secretion increased when cells were incubated in osteogenic medium. However, OPG concentration was lower in 3D than in 2D cultures. This effect was observed in both osteogenic and growth media and resulted in slightly higher RANKL to OPG molar ratios in hMSC cultured in 3D than on 2D substrates (Fig. 5, upper-right panel). Macrophage colony-stimulating factor (M-CSF) secretion increased when hMSC were incubated in medium containing inducers of osteoblast differentiation. In growth and osteogenic media, levels were higher in 3D than in 2D cultures. In the absence of differentiating factors, hMSC grown in 3D substrates secreted lower levels of IL-6. Regardless of the type of substrate, cells switched to the osteoblastic phenotype largely diminished the secretion of IL-6. Substrate type did not interfere with VEGF secretion, which was substantially lower in cultures incubated in osteogenic medium. Levels of VEGFR-1 were significantly lower in 3D than in 2D cultures. In both cases, osteogenic medium stimulated the secretion of the receptor.

**Figure 6:**
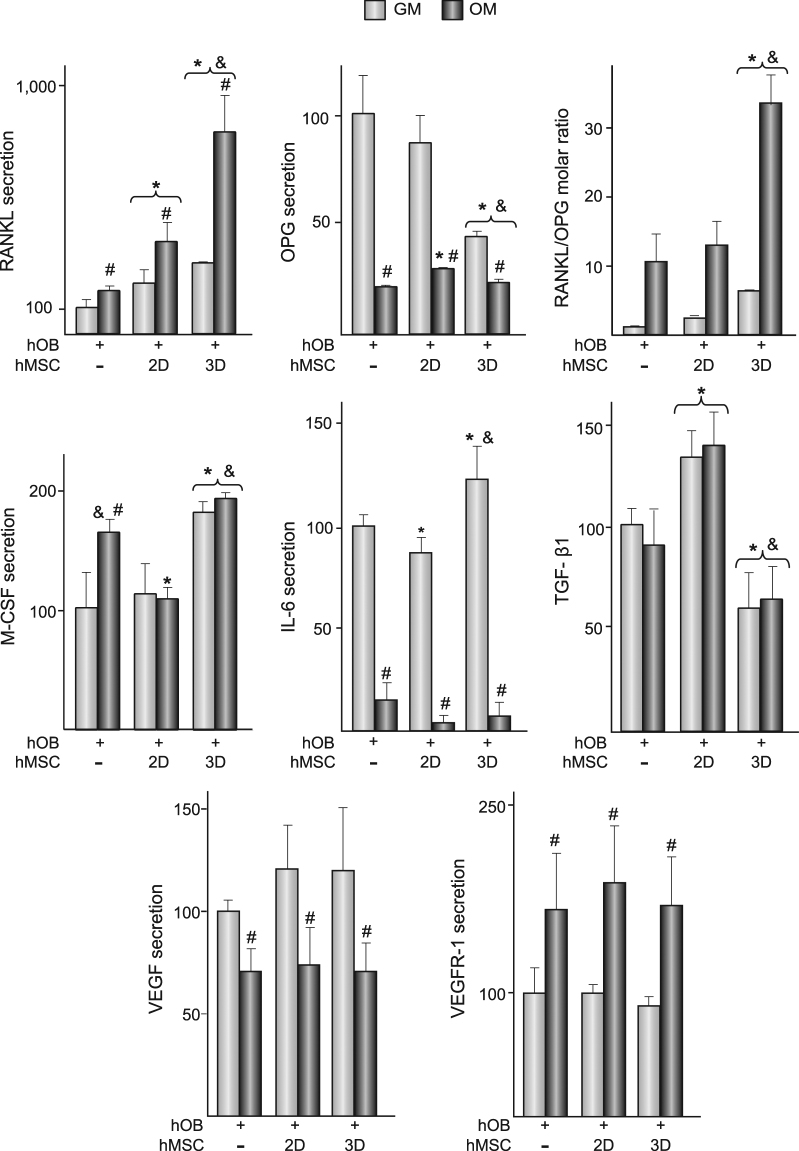
RANKL, OPG, M-CSF, IL-6, VEGF and VEGFR-1 secretion, and TGF-β1 production in co-cultures of hOB and hMSC. hOB were cultured in isolation (−) or co-cultured with hMSC seeded on 2D or 3D substrates up to 14 days, in growth (GM) or osteogenic (OM) media. Protein levels are relative to those measured in hOB cultured in isolation in GM, which were given an arbitrary value of 100 and correspond to the values shown in the Table 2. TGF-β1 data are relative to the levels quantified in cell layers of isolated cultures of hOB, which were given an arbitrary value of 100 and correspond to 40 ± 3 ng mg protein^−1^. *p < 0.05 compared with isolated cultures of hOB, at the corresponding experimental condition. ^#^p < 0.05 compared with cells cultured in GM. ^&^p < 0.05 compared with hOB co-cultured with hMSC seeded on 2D substrates, at the corresponding experimental condition.

A final set of experiments explored whether the levels of the soluble factors previously examined in cultures of hMSC grown on  the substrates (Fig. 5) are differentially modulated in co-cultures of hOB and hMSC (Fig. 6). When cultured in growth medium, RANKL secretion by hOB was about 20 times lower than in cultures of hMSC grown on 2D substrates while osteoblastic OPG secretion was about 60-fold higher (Table 2). Consequently, the RANKL to OPG molar ratio in hOB cultures was around one thousand-fold lower than in hMSC cultures. Cultures of hOB and co-cultures contained higher RANKL levels in osteogenic than in growth media (Fig. 6, upper-left panel). In both media, co-culturing induced an increase in RANKL levels, which was higher in 3D co-cultures. Growth medium from 2D co-cultures contained similar amounts of OPG than medium from isolated cultures of hOB (Fig. 6, upper-center panel). However, co-culturing hOB with hMSC seeded in 3D substrates resulted in an important decrease in the levels of the decoy receptor. OPG levels were lower in isolated cultured hOB and in co-cultures incubated in osteogenic than in growth media, which correlated with an important increase in RANKL to OPG molar ratio. Ratios were higher in 3D than in 2D co-cultures (Fig. 6, upper-right panel). We then examined the modulation of M-CSF and IL-6 secretion in 2D and 3D co-cultures. When incubated in growth medium, hOB secreted higher amounts of M-CSF than hMSC (Table 2). Incubation in osteogenic medium stimulated the osteoblastic secretion of the factor. When hOB were co-cultured in growth medium with hMSC seeded on flat substrates, M-CSF levels remained as in isolated cultures of hOB. However, the concentration of M-CSF increased in 3D co-cultures incubated in growth medium. No increase in M-CSF levels was detected when co-cultures were incubated in osteogenic medium. When cultured in growth medium, hOB produced higher amounts of IL-6 than hMSC (Table 2). Co-culturing with hMSC seeded on 2D substrates slightly decreased IL-6 secretion which increased in 3D co-cultures. Incubation in osteogenic medium led to an important decrease in IL-6 levels in hOB cultured in isolation or in the co-cultures. Levels of transforming growth factor beta-1 (TGF-β1) in hOB monolayers, cultured in isolation or co-cultured, were not affected by the type of medium. Compared with hOB cultured in isolation, the content of TGF-β1 significantly increased in 2D co-cultures while decreased in 3D co-cultures. No significant differences in VEGF secretion were detected in hOB and hMSC cultured on 2D substrates in growth medium (Table 2). Incubation in osteogenic medium reduced the osteoblastic secretion of VEGF. hOB cultures secreted substantially higher amounts of VEGFR-1 than hMSC (Table 2). As observed in hMSC (Fig. 5), incubation in osteogenic medium resulted in increased osteoblastic secretion of VEGFR-1. Regardless of the type of substrate, co-culturing did not affect the levels of VEGF or VEGFR-1 secreted by hOB.

## Discussion

Numerous studies comparing the responses of cells cultured on flat substrates or in three-dimensional biomaterials provide evidence that the microstructural features of the substrates have a profound impact on the functionality of many adherent cell types, including MSC. Stiffness, topography, texture and mechanical properties of the substrates influence self-renewal, fate commitment and secretory profile of MSC^18,19^. However, the roles that the structural properties of the substrates exert to finely regulate cell-to-cell communications have not been fully elucidated yet. Having recently shown that topographical cues modulate the paracrine immunomodulatory ability of hMSC^17^, data herein provide evidence that the crosstalk between these cells and cell types involved in bone regeneration, namely hOB and hEC, is also regulated by substrate microarchitecture.

Incubation in growth medium impeded the development of capillary-like structures in HUVEC cultures, likely due to the high concentration of glucose in this medium, which impairs *in vitro* angiogenic activity^20^. Interestingly, growth medium conditioned by hMSC cultured on three-dimensional scaffolds stimulated the tubular reorganization of HUVEC, an effect greatly diminished in medium conditioned by hMSC cultured on flat substrates. Impaired endothelial function by elevated glucose has been related to lower expression of VEGF^21^ and angiopoietin-1^20^ although secretion of these factors from hMSC was not sensitive to the topography of the substrates employed in our study. However, levels of the VEGF-sequestering receptor VEGFR-1 were lower in conditioned medium from hMSC cultured in 3D than on 2D substrates, which suggests that three-dimensionality may favour the stimulation of hEC functions by increasing the availability of VEGF. Modulation of the levels of proangiogenic MCP-1 and IL-6^22^ does not seem to contribute to the stimulation of tubular reorganization of HUVEC as their secretions were substantially lower in cultures of hMSC incubated on 3D than on 2D substrates, as previously detected in the same materials^17^. IGF-1, which enhances the vessel-forming capacity of hEC^23^, was secreted at remarkably higher extent in medium conditioned by cells grown in 3D scaffolds, thereby likely contributing to HUVEC activity. Local administration of conditioned medium by hMSC grown on standard culture plastic promotes angiogenesis in bone defects, as shown in a mouse model of distraction osteogenesis^24^ and in a fracture healing model in diabetic rats^25^. In view of results herein, conditioned medium from hMSC cultured in 3D scaffolds might contain factors that enhance *in vivo* vessel formation at a higher extent than those obtained from cells cultured on conventional flat surfaces.

The high content of glucose in growth medium is not only detrimental to the ability of HUVEC to form capillary-like structures but also impairs its viability^20,21^. Interestingly, co-culturing with hMSC greatly increase metabolic and migratory activities of HUVEC. The contents of potent growth factors are differentially modulated in 2D and 3D co-cultures, suggesting that hMSC might induce angiogenic activity through paracrine mechanisms that are sensitive to the substrate microarchitecture. Crosstalk between HUVEC and hMSC lowered the levels of IGF-1 and EGF, although the decrease was less pronounced when hMSC were grown in three-dimensional substrates. FGF-2, another main regulator of angiogenesis, could only be detected in 3D co-cultures. Thus, structural complexity of the scaffold may tune the interplay between the two cell types towards higher production of IGF-1, EGF and FGF-2, resulting in increased proliferation and migration of hEC. Levels of IL-6, which are higher in co-cultures than in isolated cultures of HUVEC, were also sensitive to the topography of the substrates that harbour hMSC. It is worth noting that the levels of some proteins, as angiopoietin-1, IGF-1 or IL-6, are modulated by the substrates following similar trends in isolated cultures of hMSC and in co-cultures. However, the microarchitecture of the substrate affects the levels of VEGFR-1 and MCP-1 in isolated cultures of hMSC but not in the co-cultures. Thus, secretion data obtained from hMSC cultured in isolation on biomaterials should be extrapolated cautiously as, upon implantation, *in vivo* crosstalk with other cell types may result in rather different secretion outcomes. A surprising finding of our study is that VEGF could not be detected in co-culture media whereas hMSC cultured in isolation secrete substantial amounts. It cannot be excluded that paracrine interactions with HUVEC inhibit VEGF secretion in co-cultured hMSC. However, having into account the essential role that VEGF plays in EC proliferation and survival, a more plausible possibility is that secreted VEGF from co-cultured hMSC binds to fibronectin in the HUVEC extracellular matrix, retaining functional activity^26^. In fact, the extracellular matrix may not only sequester and store soluble growth factors but also present them to their cell receptors^27^.

Metabolic activity of hMSC was markedly diminished in 3D substrates. The possibility that the decrease was due to impaired cell viability was excluded since cells can populate 2D and 3D substrates at a similar extent. Oxidative metabolism, glycolysis and mitochondrial biogenesis govern stemness and differentiation pathways of MSC and are influenced by their microenvironment, including changes in metabolites concentration or O_2_ pressure^28^. To our knowledge, it remains unknown whether substrate features regulate hMSC metabolism. Data herein suggest that this could be the case. Osteogenic differentiation of hMSC can be influenced by host bone-forming cells, as reported in previous *in vitro* studies^14,29^. Our results indicate that the architecture of the substrate may play an important role in such remote control. Thus, interactions with terminally differentiated hOB stimulate ALP activity of stromal cells seeded on 3D substrates but not on 2D substrates. Interestingly, osteoblastic ALP activity and PICP levels increase in co-cultures with hMSC. Implantation of a collagen sponge seeded with hMSC in a calvaria defect in rats significantly increased host *Col1a1* and *Alpl* expression^30^. We have detected that the microarchitecture of the substrates that harbour hMSC influences *COL1A1* and *ALPL* expression levels in human bone-forming cells, suggesting a positive effect of the three-dimensionality on early steps of host osteoblastic matrix reorganization. However, specific osteoblastic functions seemed to be demoted by co-culturing with hMSC, as degree of mineralization and *BGLAP* mRNA levels were lower in co-cultures, and more markedly, in 3D co-cultures.

The bone regenerative activity of factors secreted by hMSC has been shown after local administration of conditioned medium in bone defects^4,24,25^. All available data on the effect of conditioned media from MSC on bone healing have been obtained by culturing these cells in standard, flat, tissue culture plastic. Data herein encourage the search for culture conditions that take into account the structural features of the culture substrate, i.e. three-dimensional geometry, to tune the secretomic profile of hMSC towards optimal therapeutic profile.

Tissue intermediates designed for bone tissue engineering need to provide support for the dynamics of bone remodelling, i.e. promote bone formation by host osteoblasts and then be slowly resorbed by host osteoclasts^31^. hMSC and bone forming cells, among other cell types, control osteoclastogenesis and bone resorption through the production of RANKL and OPG. The latter inhibits bone resorption by disrupting the interaction between RANKL and RANK, which is expressed on the surface of osteoclasts and their progenitors^32^. Thus, increased RANKL to OPG ratios favour bone turnover. Co-culturing hMSC and hOB increases the molar ratio RANKL to OPG, but the increase is substantially higher in 3D than in 2D co-cultures, both in growth and osteogenic media. M-CSF, an essential factor that promotes the expression of RANK in osteoclasts precursors and enables them to respond to RANKL for further differentiation along the osteoclastic lineage^33^, was also sensitive to the substrate. Not only M-CSF levels were higher in isolated cultures of hMSC incubated in three-dimensional than on flat substrates but also higher in 3D than in 2D co-cultures. The levels of IL-6, which amplifies the effects of pro-resorptive agents through paracrine and autocrine mechanisms and collaborates in the production of pro-resorptive factors^34^, were also higher in 3D than in 2D co-cultures. Low concentrations of TGF-β1 stimulate osteoclast recruitment, development and survival while high concentrations inhibit osteoclastogenesis and promote osteoclast apoptosis^35^. Decreased levels of TGF-β1 were detected in the osteoblastic cell layer of 3D co-cultures, suggesting that scaffold microarchitecture might enhance osteoclast activity by modulating the levels of this factor. Altogether, these results suggest that the architectural features of the substrate might be a decisive parameter that controls bone remodelling around the tissue intermediate.

## Methods

### Materials

Tissue culture plastic manufactured from polystyrene was purchased to Corning. Highly-porous (>90%) scaffolds of cross-linked polystyrene (Alvetex^®^, Reprocell), referred to as “3D substrates”, were manufactured using an emulsion template technique to control the size of the pores^36^. Following manufacturer’s instructions, 3D substrates were immersed in 70% ethanol for 5 min and then thoroughly washed with phosphate buffered saline (PBS) before cell seeding. Polyester cell culture inserts (Corning), referred to as “2D substrates”, were used as flat permeable supports. Topographical characterization of the investigated substrates was assessed by means of scanning electron microscopy (SEM) using an FEI Quanta 200 Environmental scanning electron microscope (Fig. 1a). Pore size distribution in the substrates was analyzed in the course of our previous work^17^. 2D substrates present a homogenous pore size distribution with a mean diameter of 0.51 µm while 3D substrates show randomly oriented and interconnected pores which diameters range between 2 to 70 µm (90% pores with diameters lower than 20 μm). The average surface roughness (R_a_) of flat supports was 7.2 nm for tissue culture plastic and 4.7 nm for cell culture inserts, as determined using an optoelectronic profilometer (p-6 Stylus Profiler, KLA-Tencor Corporation).

### Cell culture

Bone marrow-derived hMSC (Lonza) were expanded in a defined medium consisting of MSC basal medium and SingleQuots growth supplements (Lonza). hMSC were tested routinely by means of flow cytometry for the presence of MSC-associated surface molecules CD105, CD29, CD44, and the absence of hematopoietic markers CD14, CD34 and CD45 (data not shown). Experiments were carried out with hMSC cultured up to 7th passage. HUVEC were purchased from Lonza and expanded in endothelial cell growth medium (EGM-2, Lonza) in flasks pre-coated with 0.1% (w/v) gelatin from bovine skin (Sigma-Aldrich) in PBS. Experiments were carried out with HUVEC cultured up to 6th passage. Primary hOB were obtained from trabecular bone explants aseptically collected from patients undergoing total orthopedic knee arthroplasty as previously described^37^. Each bone sample was processed in a separated culture and experiments were performed using independent cultures obtained from 12 patients. For subsequent experiments, confluent cultures were subcultured from initial isolates. Experiments were carried out with hOB cultured up to the 2nd passage. Patients enrolled in this research signed an informed consent form. All procedures using human tissue designated “surgical waste” were approved by the Human Research Committee of University Hospital La Paz (Date of Approval: 07/11/2012). All experiments and methods were performed in accordance with relevant guidelines and regulations. Bone fragments were cultured in growth medium consisting of Dulbecco’s Modified Eagle’s Medium containing 4.5 gL^−1^ glucose (Lonza) supplemented with 15% (v/v) heat inactivated fetal bovine serum, 10 U mL^−1^ penicillin and 0.01 mg mL^−1^ streptomycin.

Cells were maintained in a humidified 5% CO_2_ atmosphere at 37 °C.

### Co-cultures of hMSC and HUVEC or hOB

hMSC were co-cultured with HUVEC or hOB using a cell culture insert that allows paracrine communication of both cell types and prevents direct contact between co-cultured cells. HUVEC were seeded at a density of 10^4^ cells cm^−2^ on 6-well plates pre-coated with 0.1% (w/v) bovine gelatin in PBS, and cultured in EGM-2 for 1 day. hOB were seeded at a density of 10^4^ cells cm^−2^ on 6-well plates and cultured in growth medium for 1 day. hMSC were seeded at a density of 10^4^ cells cm^−2^ directly on the inserts or in 3D substrates placed on the inserts, and then cultured in growth medium for 1 day. Inserts harboring hMSC were washed with PBS and placed into the wells containing hOB or HUVEC. Growth medium was added to co-cultures to reach a final volume of 3 mL. Co-cultures of hMSC and HUVEC or hOB were further incubated up to 7 or 14 days, respectively. In some experiments, co-cultures of hMSC and hOB were incubated for 14 days in osteogenic medium consisting of growth medium supplemented with 10^−7^ M dexamethasone, 3 × 10^−4^ M ascorbic acid and 10^−2^ M β-glycerophosphate. In co-cultures maintained for 14 days, medium was partially replaced with an equal volume of fresh medium after 7 days of culturing, to prevent nutrient exhaustion. As controls, hOB and HUVEC were incubated without co-culturing with hMSC. In some experiments, hMSC were incubated for 7 or 14 days on 2D or 3D substrates without co-culturing with HUVEC or hOB.

### Immunofluorescence assays

Cells were washed with PBS followed by fixation in 4% (w/v) paraformaldehyde in PBS and permeabilization with 0.1% Triton X-100 in PBS. Cells were blocked in 2% bovine serum albumin (BSA) in PBS containing 0.05% Tween 20 and then incubated with mouse anti-human fibronectin monoclonal antibody (Chemicon) diluted 1:100 in 1% BSA in PBS. Cells were washed with 0.05% Tween 20 in PBS followed by incubation with goat anti-mouse Alexa-Fluor 488 (Molecular Probes) diluted 1:1000 in 1% BSA in PBS. To label actin cytoskeleton, cells were additionally incubated with PBS containing 4 × 10^−7^ M phalloidine-TRITC. After washing with PBS containing 0.05% Tween 20, cells were imaged using a confocal microscope (Leica TCS SPE). Image area occupied by actin or fibronectin was quantified using ImageJ v1.34 image analysis software (https://imagej.nih.gov/ij/).

### Metabolic activity assays

Metabolic activity of hMSC, HUVEC or hOB was assessed as a cell viability index using the alamarBlue assay (Biosource), as previously described^37^. Metabolic activity was measured immediately before setting the co-cultures, 3 and 7 days after co-culturing hMSC and HUVEC, or 7 and 14 days after co-culturing hMSC and hOB.

### Immunoenzymatic assays

Cultured media were collected and centrifuged at 1,200 g for 5 min. Media were supplemented with 17.5 μg mL^−1^ phenyl-methylsulfonylfluoride, 1 μg mL^−1^ pepstatin A, 2 μg mL^−1^ aprotinin and 50 μg mL^−1^ bacitracin, and stored at −80 °C. Human specific ELISA kits were used to measure angiopoietin-1, MCP-1, FGF-2, EGF, IGF-1, VEGF, VEGFR-1, PICP and IL-6 (all from R&D Systems), OPG (Bender MedSystems GmbH), RANKL (Biomedica Gruppe) and M-CSF (Abcam). For TGF-β1 detection, cell layers were washed with PBS and extracted with 5 × 10^−2^ M Tris-HCl pH 8.0, 5 × 10^−1^ M NaCl and 1% Triton X-100, supplemented with protease inhibitors as above described. The amount of TGF-β1 in the extracts was determined using a human specific ELISA (RayBiotech) and the data were normalized to the total protein amount in cell layers, as determined by a Bradford-based protein assay (Bio-Rad Laboratories Inc.).

### Endothelial cell tube formation assay

hMSC were seeded on 2D or 3D substrates at a density of 10^4^ cells cm^−2^ and cultured for 7 days in growth medium. Media were collected, centrifuged at 1,200 g for 5 min and used immediately for the endothelial cell tube formation assays. For this purpose, confluent HUVEC were cultured for 24 h in endothelial basal medium (EBM, Lonza) supplemented with 10 U mL^−1^ penicillin and 0.01 mg mL^−1^ streptomycin. Then, HUVEC were trypsinized and resuspended at a density of 1.25 × 10^5^ cells mL^−1^ in EGM-2, in growth medium, or in growth media conditioned by hMSC cultured for 7 days on 2D or 3D substrates. One mL of HUVEC suspension was seeded on 24-well plates containing 280 µl of growth factor reduced Matrigel (GFR-Matrigel, Corning). After incubation for 18 h, cells were examined using an inverted microscope.

### Wound healing assay

HUVEC were seeded on 6-well plates at a density of 10^4^ cells cm^−2^ in EGM-2. Once cells reached confluence, medium was replaced with EBM supplemented with 10 U mL^−1^ penicillin and 0.01 mg mL^−1^ streptomycin. In parallel, hMSC were seeded on 2D or 3D substrates and cultured in growth medium. After 24 h, HUVEC monolayers were washed with PBS and scratched using a sterile 200 µl pipette tip. Detached cells were removed by extensive PBS washings. Inserts harbouring hMSC cultured on 2D or 3D substrates for 24 h were placed in wells containing HUVEC and growth medium was added to co-cultures to achieve a final volume of 3 mL. As controls, HUVEC cultures were scratched but incubated in growth media without co-culturing with hMSC. Phase contrast images were taken immediately after scratching the HUVEC monolayers and after culturing for 12 h, using an inverted microscope. Invaded area was measured using ImageJ v1.34 image analysis software.

### ALP activity and cell layer calcification assays

Cell layers were washed with PBS and extracted as detailed above. ALP activity was determined in the extracts by determining the release of *p*-nitrophenol from *p*-nitrophenylphosphate at 37 °C and pH 10.5. The data were normalized to the total protein amount in cell layers.

The degree of mineralization of cell layers was determined using Alizarin Red staining. Briefly, cells were fixed with ethanol and stained with 40 mM Alizarin Red in deionized water at pH 4.2. The bound stain was eluted with 10% (w/v) cetylpyridinium chloride and the absorbance at 562 nm was measured using a Synergy4 multi-mode microplate reader.

### Analysis of differential gene expression

Total RNA was isolated using TRI Reagent (Molecular Research Center, Inc.). Complementary DNA was prepared from total RNA with Transcriptor First Strand cDNA Synthesis Kit using an anchored-oligo (dT)18 primer (Roche Applied Science). Real-time quantitative PCR was performed using LightCycler FastStart DNA Master SYBR Green I and LightCycler instrument (both from Roche Applied Science). Quantitative expression values were extrapolated from standard curves, and normalized to β2-microglobulin (*β2 M*) values. Specific oligonucleotide primers were: *COL1A1*, 5′-CGGGCCTCAAGGTATTGCT-3′ (forward primer, F) and 5′-GGGACCTTGTTTGCCAGGTT-3′ (reverse primer, R); *ALPL*, 5′-GACTAAGAAGCCCTTCACTGCCAT-3′ (F) and 5′-GACTGCGCCTGGTAGTTGTT-3′ (R); *BGLAP*, 5′-GGCGCTACCTGTATCAATGG-3′ (F) and 5′-GATAGGCCTCCTGAAAGCCG-3′ (R); *β2* *M*, 5′-CCAGCAGAGAATGGAAAGTC-3′ (F) and 5′-GATGCTGCTTACATGTCTCG-3′ (R).

### Statistical analysis

The data are presented as means ± S.D. of at least five independent experiments. Quantitative data were tested using Mann-Whitney U rank-sum and two-sided Kruskal-Wallis tests. *Post hoc* comparisons were analyzed by the Mann-Whitney U test, adjusting the p value with the Bonferroni correction, and the level of significance was set to p < 0.05. All statistical analyses were performed using the Statistical Package for the Social Sciences, version 15.0 (SPSS Inc.).
